# Detecting MRI-Invisible Prostate Cancers Using a Weakly Supervised Deep Learning Model

**DOI:** 10.1155/2024/2741986

**Published:** 2024-03-19

**Authors:** Yao Zheng, Jingliang Zhang, Dong Huang, Xiaoshuo Hao, Weijun Qin, Yang Liu

**Affiliations:** ^1^School of Biomedical Engineering, Air Force Medical University, No. 169 Changle West Road, Xi'an, Shaanxi, China; ^2^Department of Urology, Xijing Hospital, Air Force Medical University, No. 127 Changle West Road, Xi'an, Shaanxi Province, China

## Abstract

**Background:**

MRI is an important tool for accurate detection and targeted biopsy of prostate lesions. However, the imaging appearances of some prostate cancers are similar to those of the surrounding normal tissue on MRI, which are referred to as MRI-invisible prostate cancers (MIPCas). The detection of MIPCas remains challenging and requires extensive systematic biopsy for identification. In this study, we developed a weakly supervised UNet (WSUNet) to detect MIPCas.

**Methods:**

The study included 777 patients (training set: 600; testing set: 177), all of them underwent comprehensive prostate biopsies using an MRI-ultrasound fusion system. MIPCas were identified in MRI based on the Gleason grade (≥7) from known systematic biopsy results.

**Results:**

The WSUNet model underwent validation through systematic biopsy in the testing set with an AUC of 0.764 (95% CI: 0.728-0.798). Furthermore, WSUNet exhibited a statistically significant precision improvement of 91.3% (*p* < 0.01) over conventional systematic biopsy methods in the testing set. This improvement resulted in a substantial 47.6% (*p* < 0.01) decrease in unnecessary biopsy needles, while maintaining the same number of positively identified cores as in the original systematic biopsy.

**Conclusions:**

In conclusion, the proposed WSUNet could effectively detect MIPCas, thereby reducing unnecessary biopsies.

## 1. Introduction

Prostate cancer (PCa), increasingly diagnosed in men, significantly threatens male health worldwide [1–3], and pathological biopsy stands as the definitive diagnostic tool for PCa [4]. Currently, MRI-ultrasound fusion-targeted biopsy improves the positive detection rate compared with systematic biopsy [5]. However, the imaging appearances of some PCas are similar to those of the surrounding normal tissue on magnetic resonance imaging (MRI) [6]. These hard-to-spot cases are referred to as MRI-invisible prostate cancers (MIPCas) [6, 7].

Recent clinical studies highlight the indispensable role of systematic biopsies in identifying MRI-invisible prostate cancers [8–10]. A study published in the New England Journal of Medicine [11] found that systematic biopsies for MRI-invisible lesions resulted in a diagnostic upgrade in 9.9% of patients, in contrast to targeted biopsies for MRI-visible lesions. These findings highlight the urgent need for enhanced accuracy in detecting and diagnosing MIPCas, emphasizing the significant impact and relevance of our research in the realm of MRI-invisible prostate cancer. Due to the MRI-invisible appearance, systematic biopsy detection rates are restricted. Therefore, enhancing the detection accuracy of MIPCas in MRI is of utmost importance.

In recent years, the role of artificial intelligence has grown in cancer detection via MRI scans, bringing about pivotal advancements [12–15]. However, conventional supervised detection algorithms rely on detailed lesion delineation by radiologists. Due to the MRI-invisible appearance of MIPCas, it is difficult to outline these lesions [16, 17]. Weakly supervised learning is a method that uses partially labelled data to learn the whole distribution [18, 19]. These advances provide a new opportunity to detect MIPCas using biopsy data.

Against this backdrop, our study presents a unique tactic that combines deep learning with weakly supervised training to enhance MIPCa detection. We hypothesized that deep learning networks performing weakly supervised training would capture potential cancer imaging features to detect MIPCas.

## 2. Materials and Methods

### 2.1. Patient Enrolment

Our analysis was performed in an openly accessible dataset (Prostate-MRI-US-Biopsy) [20, 21]. This dataset includes biopsy sessions carried out using the Artemis system, which integrates real-time ultrasound with preoperative MRI to collect biopsy samples from regions of interest identified in preoperative MRI. Additional systematic biopsy samples were obtained via a digital template. The Artemis system recorded all biopsy core locations in relation to the MRI. The dataset comprises patients who were suspected to have prostate cancer due to high PSA levels and/or suspicious imaging findings and underwent—or planned to undergo—routine standard-of-care prostate biopsies at the UCLA Clark Urology Center. T2-weighted MRI, specific biopsy core locations, Gleason grade, and clinical information (including lesion outline, PSA, and PI-RADS) formed the core components of our analysis.

**Figure 1:**
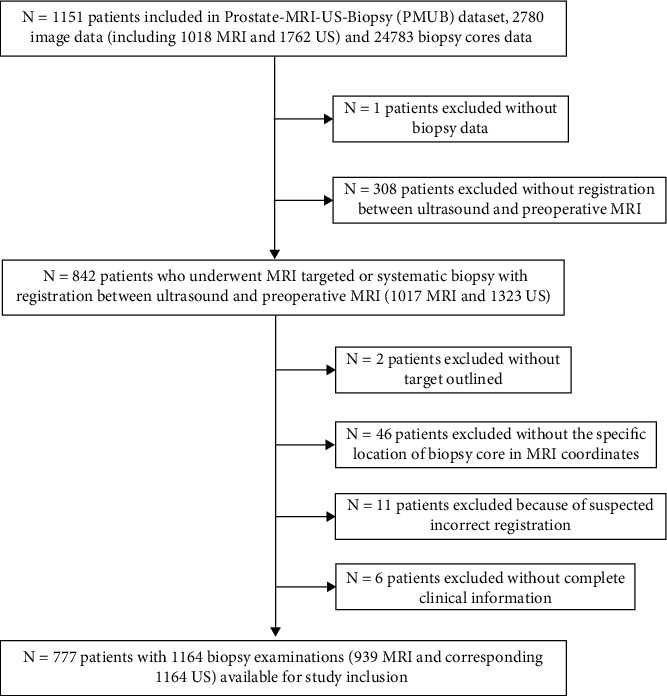
Flowchart showing participant inclusion and data partitioning.

The dataset originally contained 1151 patients, of which 777 met the inclusion and exclusion criteria as outlined in Figure 1. The exclusion criteria were as follows: *N* = 1patient was excluded due to the lack of biopsy data; *N* = 308 patients without registration between ultrasound and preoperative MRI were excluded; *N* = 2 patients without target outlines were omitted; *N* = 46 patients lacking specific biopsy core location in MRI coordinates were eliminated; *N* = 11 patients due to suspected inaccurate registration were removed; and *N* = 6 patients were excluded for incomplete clinical information.

### 2.2. Identification of MRI-Invisible Prostate Cancers (MIPCas)

#### 2.2.1. MRI Analysis and Lesion Delineation

Initially, all patients underwent multiparametric MRI, which included T2-weighted imaging, diffusion-weighted imaging (DWI), and perfusion-weighted imaging (PWI). Each mpMRI scan was meticulously reviewed by prostate radiologists who delineated all visible lesions (ROIs), irrespective of their perceived malignancy potential.

#### 2.2.2. Biopsy Procedure

After MRI analysis, patients received both targeted biopsies (for delineated ROIs) and systematic biopsies. This integrated biopsy approach was designed to detect not only MRI-visible lesions but also those MRI-invisible lesion. Acknowledging the limitations of ROIs in cancer detection, this strategy was especially tailored to address MRI-invisible cancers, reducing potential cancerous lesion.

#### 2.2.3. Determination of MIPCas

**Figure 2:**
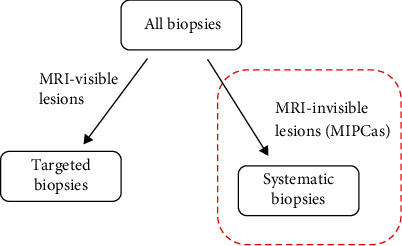
Determination of MIPCas.

The identification of MIPCas was based on a combined evaluation of biopsy outcomes and MRI findings. Specifically, as depicted in Figure 2, MIPCas were primarily detected through systematic biopsies of areas not characterized as ROIs on mpMRI, considering the potential for mpMRI to miss or inaccurately characterize some cancers.

### 2.3. Deep Learning Model

In this research, we propose an innovative concept, the weakly supervised UNet (WSUNet). This model is fundamentally based on a 3D UNet framework [22]. Our key objective was to effectively extract salient features from T2-weighted MRI data, synergize it with relevant clinical information, and subsequently create comprehensive 3D cancer region probability distribution maps. These maps serve as an invaluable tool in the diagnostic process, design of treatment strategies, and monitoring of disease progression. This anchors our WSUNet as an important tool within patient management and care.

### 2.4. Weakly Supervised Module

To enable our model to learn to detect MIPCas from biopsy data, we introduced a weakly supervised module. This module was formulated around the strategic integration of crucial biopsy location data and Gleason grading, where a score of 7 and above was marked as positive biopsy. Based on the macroscopic spatial location information of the biopsy and the microscopic pathological information of Gleason grade, the model can search for MIPCas missed by radiologists on the whole prostate MRI.

**Figure 3:**
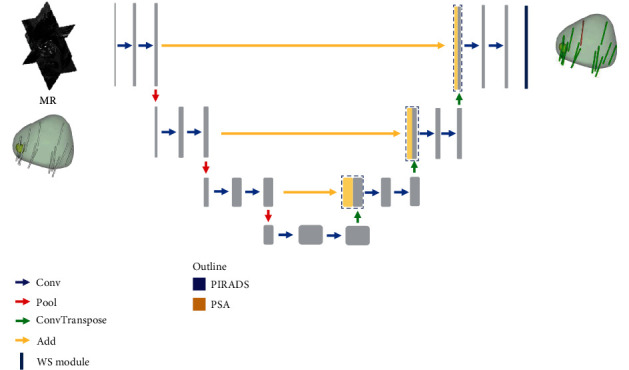
The main structure of WSUNet. Conv, Pool, ConvTranspose, and WS module are layers for feature extraction. Add refers to the addition of feature maps.

This weakly supervised module unifies two core operational elements: an interpolation operation and a maximum pooling operation. The interpolation operation symbolizes the sampling process for each biopsy. On the other hand, the maximum pooling operation is put into action to facilitate multi-instance learning [23], where the presence of any positive instances within the biopsy core results in the classification of the entire core as positive. These decision-making elements align with clinical biopsies, allow for an accurate representation of the biopsy, and enhance the model's understanding of MIPCas. Additionally, a simple decision tree assists in obtaining patient-level output weighting. To counteract imbalances in our data, we have employed weighting of loss functions and calibration of output probabilities. The primary structure of the model is depicted in Figure 3.

### 2.5. Weakly Supervised Framework

**Figure 4:**
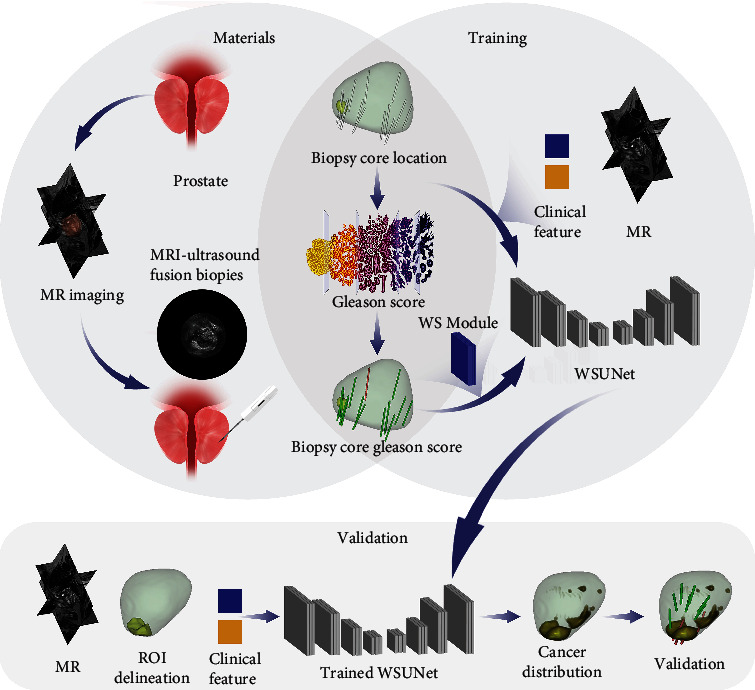
The workflow for material collection, model training, and validation. *Materials* included T2-weighted MRI, MRI location, Gleason grade of each biopsy, and the outline of the lesion and prostate, which were outlined by a radiologist (the delineation of lesions and the MRI-invisible appearance of MIPCas were both based on multimodal MRI), and clinical features, which include PI-RADS of the outlined lesions and the patient's prostate-specific antigen. *Training*: the model was trained to classify whether the Gleason grade was greater than or equal to 7 for each biopsy core to obtain the ability to correlate spatial location with pathological information. *Validation*: the model generates cancer distribution maps for each MRI in the testing set, and these maps were validated by systematic biopsy.

Our model training and validation process followed the sequence outlined in Figure 4: first, the model was trained to predict the Gleason grade of biopsy (grade ≥ 7) in the training set, which allowed the model to correlate spatial location and the probability of MIPCas. Therefore, the model learns completely from the location and grade information of each biopsy, without any prior knowledge or misdirection of the radiologist. The trained model was used to generate a probability map of cancer distribution in the whole prostate based on MRI. Then, to verify the performance, the model was used to generate 3D maps of MIPCas, and the maps were evaluated with systematic biopsy in the testing set.

The deep learning networks and overall framework were implemented using PyTorch [24] (version 1.12; https://pytorch.org/) backend in Python (version 3.9.16; Python Software Foundation) and trained with NVIDIA A100 (80 GB). Additional information and our code are accessible on GitHub at the following URL: https://github.com/Zhengyao0202/weakly_unet_prostate.

### 2.6. Methodological Distinction in Model Training and Validation Phases

In our study, we differentiate between the model validation and training phases, especially concerning the treatment of biopsy needles overlapping with ROIs. In the validation phase, all biopsies that overlapped with the ROI, whether systematic or targeted, were excluded to ensure the accuracy of the assessment and to eliminate bias. This exclusion prevents the model from being falsely credited for merely identifying lesions associated with biopsy sites.

Conversely, for the training phase, we did not exclude biopsy data that overlapped with ROIs. The rationale is that identifying visible lesions is foundational, and if a model can detect MIPCas, it should also identify obvious lesion features. Including these overlapping data points during training enriches the dataset, facilitating comprehensive model learning by covering a broader spectrum of lesion characteristics.

### 2.7. Statistical Analysis and Performance Evaluation

Homogeneity of clinical characteristics was assessed using the chi-square test and Mann–Whitney *U* test. The performances of WSUNet were measured using the receiver operator characteristic (ROC) analysis, and the area under the ROC curve (AUC) was calculated. Sensitivity and AUC were also measured via bootstrapping with 1000 resamples. We evaluated an important metric, precision, which can be considered as a special kind of detection rate, compared it with the precision of the original systematic biopsy, calculated the improvement of our method, and further calculated the number of unnecessary biopsies that can be reduced by our model. In light of multiple comparisons across our statistical analyses, we applied the Bonferroni correction to adjust the significance thresholds, setting the number of comparisons to 10. Consequently, we established a more stringent significance level at 0.005 to mitigate the risk of type I errors.

In addition, the calibration curve was plotted using the Hosmer–Lemeshow goodness-of-fit test. Decision curve analysis (DCA) was conducted to evaluate the clinical usefulness of the model by quantifying the net benefit at different threshold probabilities on both training set and testing set. We also selected some representative examples to illustrate the predictive process and advantages of our model for MIPCas.

### 2.8. Focus on MRI-Invisible Prostate Cancers (MIPCas)

To ensure our readers fully understand the focus of our study, we find it necessary to clarify that the dataset employed in our research includes results from MRI-ultrasound fusion-targeted biopsies for lesions visible on MRI, as well as systematic biopsy results for MRI-invisible lesions (MRI-invisible prostate cancers, or MIPCas). Our model's validation and testing were strictly conducted on the outcomes of systematic biopsies, meaning that our model is specifically designed to assess and test the performance exclusively on the more challenging to detect MIPCas, without considering performance on visible lesions.

Considering potential registration inaccuracies, our study emphasizes systematic biopsies performed within specific prostate zones. This methodology ensures that, even in the face of some errors, as long as these inaccuracies do not lead to mismatches beyond the designated regions, the integrity and significance of our results remain intact. Consequently, this focus enhances the robustness of our research findings.

### 2.9. Introduction of the Biopsy Saving Rate (Number)

In the evaluation of our predictive model for identifying MRI-invisible prostate cancers (MIPCas), we introduce a pivotal metric, the biopsy saving rate (number), to illustrate the efficiency improvements offered by our approach. This metric is born out of the necessity to quantify the efficacy of our model in a context sensitive to the realities of clinical practice, especially considering the retrospective nature of our study's design.

#### 2.9.1. Rationale

Our model's evaluation relies not solely on its ability to detect cancer but also on its potential to reduce unnecessary interventions. Given the retrospective design of our study, where the total number of known MIPCas is fixed, a direct comparison of the number of cancers detected between traditional systematic biopsy approaches and our model does not fully encapsulate the model's benefits. Thus, the biopsy saving rate (number) serves as an essential indicator of our model's capability to maintain high detection rates while significantly reducing the number of biopsies required—addressing a critical challenge in current prostate cancer screening practices.

#### 2.9.2. Calculation of Biopsy Saving Rate (Number)

The biopsy saving rate (number) is defined as the proportion (or number) of biopsy cores that can be avoided using our proposed model while achieving the detection of the same number of positive cores found in traditional systematic approaches. This metric is calculated as follows:

(1) $$\begin{array}{c} \text{Biopsy saving number}=\text{total number of cores sampled in systematic biopsy}-\left(\text{number of cores predicted positive by the model}*\left(\frac{\text{total positive cores in all biopsies}}{\text{number of true positive cores predicted by the model}}\right)\right),\\ \text{Biopsy saving ratio}=\frac{\text{biopsy saving number}}{\text{total number of cores sampled in systematic biopsy}}. \end{array}$$

Using this formula, the biopsy saving ratio offers a straightforward measure of efficiency improvement, reflecting how many fewer biopsy cores need to be sampled to achieve comparable positive detection outcomes. This efficiency not only speaks to the potential reduction in patient discomfort and morbidity associated with overbiopsying but also highlights the economic benefits by reducing unnecessary healthcare expenditures.

## 3. Results

### 3.1. Basic Clinical Information

In this assessment, the patient population was randomly subdivided into a training (*n* = 600) and a testing cohort (*n* = 177). As demonstrated in Table 1, the demographic and clinical features, including age, PSA levels, and the number of cores per examination, showed no significant differences between the two cohorts. *p* values for these variables all exceeded 0.05, confirming the lack of statistically significant disparities. This parity ensures that any inferential models developed can faithfully be applied from the training cohort to the testing cohort, enhancing the generalizability of this study's findings. Besides, our own dataset also reinforces the importance of this study, showing that 23.8% (433 out of 1812) of positive biopsies were from MIPCas.

### 3.2. Performance of the Proposed Models

**Figure 5:**
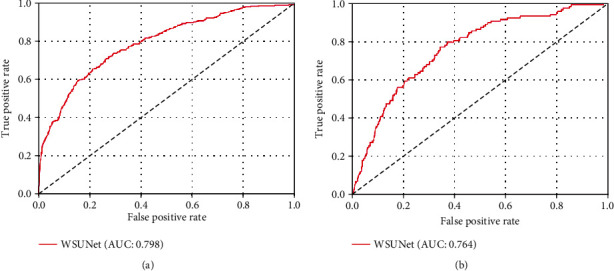
Receiver operating characteristic curves for all systematic biopsy core predictions in the (a) training set and (b) testing set.

Given the traditionally unpredictable nature of systematic biopsy outcomes for physicians, we initially considered an AUC of 0.625 as our starting point for gauging the performance of our proposed model. This baseline was established based on an analysis using the UCLA score (similar to PI-RADS v2) to predict biopsy outcomes (with Gleason score ≥ 7 as the threshold) in the all dataset. Worth mentioning, based on the filtered data, the baseline will decrease to 0.603. Nonetheless, this difference does not impact our comparative results. As displayed in Figure 5 and Table 2, the AUC of our model was recorded as 0.798 (95% CI: 0.775–0.819, *p* < 0.005) in the training set and 0.764 (95% CI: 0.728–0.798, *p* < 0.005) in the testing set, demonstrating significant improvement over the baseline.

Moreover, a model with the optimal cut-off was selected to ensure the highest levels of sensitivity and precision. The sensitivity values are depicted in Table 2, standing at 0.817 (95% CI: 0.781–0.850) in the training set and falling slightly to 0.797 (95% CI: 0.737-0.856) in the testing set. As precision substantially informs the biopsy detection rate, we assessed the enhancement in precision achieved by this model in comparison to traditional systematic biopsy. The model was found to outperform the systematic biopsy by a factor of 1.904 in the training set and 1.913 in the testing set, as shown in Table 3.

Based on these precision values, we derived a new metric termed the “sample saving rate.” This novel rate represents the fraction of biopsy cores that can be decreased using our proposed model while maintaining an equal number of positive core detections. The specific calculation method for this metric is detailed in Table 3. Consequently, our model enabled a 47.6% (*p* < 0.005) reduction in the number of biopsy needle samples in the testing set. This implies that, in the testing set, nearly half of unnecessary biopsy needles could be minimized when the positivity rate matched that of the original systematic biopsy, which is given in Table 3.

**Figure 6:**
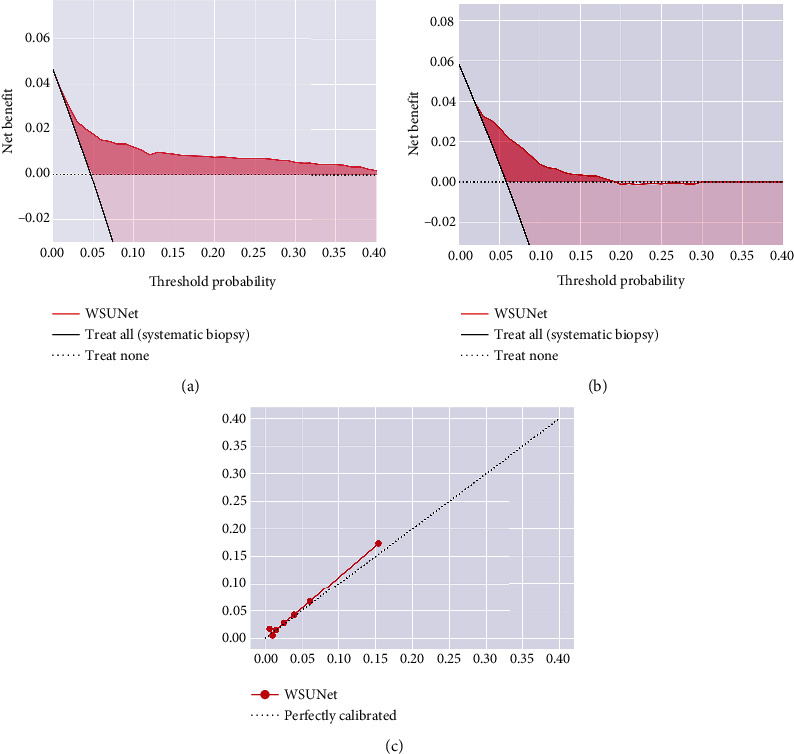
Decision curves in the (a) training set and (b) testing set. Treat All refers to the original systematic biopsy, and a large improvement can be seen for systemic biopsy, especially for high-risk patients. Treat None refers to not performing a biopsy on the patient, is considered to have a benefit of 0, and may, in fact, have some negative benefits that are difficult to evaluate. (c) Calibration curves for all data (*p* = 0.091).

**Figure 7:**
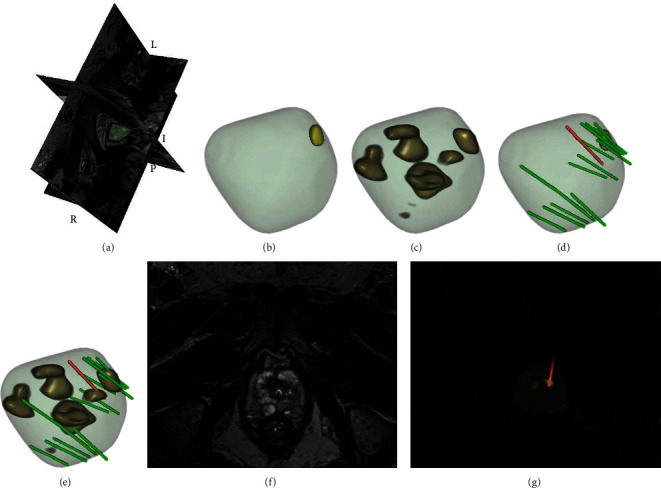
This figure presents an example of model detection: a 65-year-old man in the testing set with prostate-specific antigen of 5.5 ng/mL and PI-RADs of 4. (a) The patient's prostate is visualized in 3D coordinates for volume representation. (b) The patient's prostate surface and its ROI (yellow) outlined by radiologists. (c) The patient's prostate surface and its potential cancer distribution (dark yellow) generated by the WSUNet. (d) The patient's original ROI (yellow) and each biopsy core (Gleason grade ≥ 7 marked as red and <7 masked as green). (e) The ROI (dark yellow) detected by the model and each biopsy core (Gleason grade ≥ 7 marked as red and <7 masked as green). (f) A slice of the T2-weighted prostate MRI. (g) Slices with the detected ROI (brilliant yellow) and the positive biopsy core (red). In this meaningful example, targeted biopsy yielded no meaningful results, and the model presented a cancer distribution, part of which was validated by systematic biopsy.

**Figure 8:**
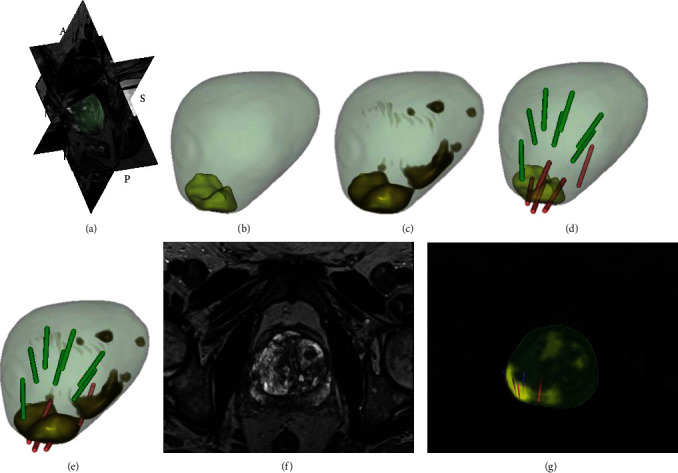
An example of model detection: a 70-year-old man in the testing set with prostate-specific antigen of 30.2 ng/mL and PI-RADs of 5. (a) The patient's prostate is visualized in 3D coordinates for volume representation. (b) The patient's prostate surface and its ROI (yellow) outlined by radiologists. (c) The patient's prostate surface and its potential cancer distribution (dark yellow) generated by the WSUNet. (d) The patient's original ROI (yellow) and each biopsy core (Gleason grade ≥ 7 marked as red and <7 masked as green). (e) The ROI (dark yellow) detected by the model and each biopsy core (Gleason grade ≥ 7 marked as red and <7 masked as green). (f) A slice of the T2-weighted prostate MRI. (g) Slices with the detected ROI (brilliant yellow) and the positive biopsy core (red). In this meaningful example, targeted biopsy yielded no meaningful results, and the model presented a cancer distribution, part of which was validated by systematic biopsy. In this example, the model successfully detected MIPCas, which radiologists have not outlined.

Our WSUNet's calibration curves showcased a consistent correlation between model-predicted positive biopsies and actual observed outcomes across all data (*p* = 0.091, as depicted in Figure 6(c)). We also performed a decision curve analysis (DCA) for each individual biopsy needle, as shown in Figures 6(a) and 6(b). The obtained curves validate the enhanced clinical benefits delivered by WSUNet compared to traditional systematic biopsy methodologies, pointing to a potential reduction in harm to the patient. Representative examples of WSUNet in comparison to conventional biopsy procedures are demonstrated in Figures 7 and 8. All of these findings lend compelling support to our initial hypothesis that a weakly supervised deep learning model can effectively discern spatial or texture attributes relevant to MIPCas.

**Figure 9:**
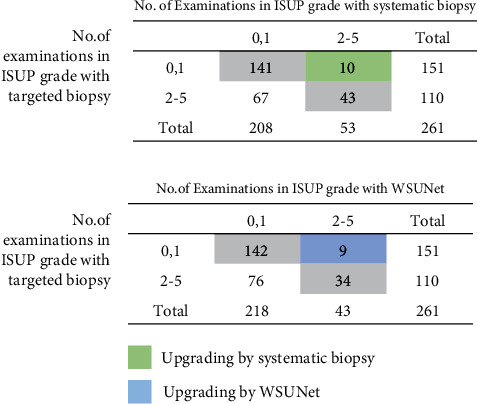
Cross-tabulation of ISUP grade group detected by biopsy method. This figure compares the outcomes of systematic and targeted biopsy, as well as the performance of our model in contrast to targeted biopsy. Green cells indicate the number of examinations upgraded through systematic biopsy, and blue cells represent those upgraded by our method (WSUNet).

We further delved into the impact of our model's limitations in fully predicting all instances of MRI-invisible prostate cancer (MIPCa). For this analysis, patients were categorized based on the International Society of Urological Pathology (ISUP) grade into two groups: ISUP 0, 1, and ISUP 2-5. Focusing on the testing set comprising 261 examinations from 177 patients, we specifically examined the upgrades in diagnosis using systematic biopsy versus targeted biopsy, as well as our method compared to targeted biopsy. The results, depicted in Figure 9, show that our model only led to a marginal decrease in the number of diagnostic upgrades (1 out of 10 examinations). It is crucial to note that this comparison may not fully represent a fair assessment, primarily due to the retrospective nature of our study, which inherently limits our ability to identify more cancers than those already known. Despite these constraints, our analysis suggests that even under these circumstances, the potential for our model to cause harm in a clinical context remains limited.

## 4. Discussion

In this study, we proposed a weakly supervised UNet (WSUNet) model for cancer detection, which represents a notable stride forward in the detection and understanding of MRI-invisible prostate cancers (MIPCas). The model demonstrated a consistent performance, achieving an AUC of 0.798 (95% CI: 0.775-0.819) in the training set and 0.764 (95% CI: 0.728-0.798) in the testing set, indicating its robustness and potential clinical utility.

Importantly, the WSUNet model has the potential to revolutionize biopsy practices. It may reduce the number of unnecessary biopsy needles by almost half, without decreasing positive detection rate. As such, WSUNet could contribute to significant improvements in patient care and follow-up, reducing the harm of each patient receives.

In comparison to existing methods, current guidelines recommend systematic biopsy due to the possibility of missed diagnoses with targeted biopsy approaches [5, 11]. Recent studies have increasingly demonstrated the power of deep learning in the detection of prostate cancer [12, 14]. However, previous detection models have been either reliant on the expertise of radiologists, which brings potential for bias and omission of MRI-invisible lesions [16, 25–27], or dependent on impractically labour-intensive manual labelling of full slice histopathology images [17, 28, 29]. As such, the WSUNet model's potential in reducing human biases and the laborious workload offers an innovative solution to these long-standing problems.

Our research was principally conducted using T2-weighted MRI data. Notably, the cancers we detected were often invisible to multimodality imaging, so these cancers may be more difficult to characterize in low-resolution functional MRI. Besides, our work has demonstrated that high-resolution T2 MRI sequences could deliver robust performance, ensuring greater clinical extensibility and broader applicability, which may be helpful for clinical extensibility and wide range of applications.

Furthermore, the field of cancer detection does not stay static. As in our previous review [30], novel imaging modalities, like prostate-specific membrane antigen positron emission tomography (PSMA PET), offer potential pathways for MIPCa detection. Despite the relatively high cost compared to T2 MRI, the expanding toolbox of imaging modalities cannot be ignored. The success of our weak supervision model lays the groundwork for its future adaptation to an array of imaging modalities, including the more precise PET imaging, heralding new potentialities for cancer diagnosis. We also consider the feasibility and potential performance improvements offered by incorporating multimodal data, such as diffusion-weighted imaging (DWI), into our analysis.

While our findings indicate promise, the retrospective nature of this study and factors such as imaging quality, physician judgment, and model deployment infrastructure highlight limitations. The true clinical utility of our model awaits further validation through prospective trials and a more diverse dataset to ensure its accuracy, effectiveness, and integration into clinical practice. Moving forward, addressing these aspects is crucial for translating our model's potential into tangible patient benefits.

A limitation of our study is the lack of detailed biopsy core locations, data not routinely available to researchers. This absence may impact our method's replicability and its broader application. Additionally, despite our best efforts to prevent it, potential registration errors may lead to overlaps between systematic biopsies and visible lesions, impacting the outcomes. Moreover, labelling all areas outside the ROIs as MRI-invisible lesions may indeed oversimplify the reality. We will work with radiologists in future experiments to better ensure that the lesions we identified are not visible.

Looking forward, the exciting performance exhibited by our WSUNet model holds potential for the future, sparking the need for wider investigations. Applying our model to large-scale prospective trials could provide more robust evidence. Furthermore, the use of multimodal imaging data, including PET/CT or follow-up data, in this model could provide more insights in understanding MIPCa detection.

In conclusion, the WSUNet model could demonstrate a promising potential in revolutionizing MIPCa detection. The results suggest that this innovative approach could make the systematic biopsy practice more accurate and patient-centric, thus reducing unnecessary biopsies while enhancing the diagnostic process's overall precision. Through conscious recognition of the model's limitations, we believe in harnessing its potential to encourage large-scale, prospective trials to improve prostate cancer detection.

## Figures and Tables

**Table 1:** Demographic and clinical characteristics of 777 included men.

Variables	Training cohort (*n* = 600)	Testing cohort (*n* = 177)	*p* value
Age^∗^	65 (61-71)	65 (61-70)	0.779
PSA^∗^	6.5 (4.4-10.1)	6.6 (4.1-9.8)	0.630
No. of core per examination^∗^	15 (12-17)	15 (12-17)	0.645
No. of systematic core per examination^∗^	10 (3-11)	10 (3-12)	0.709
No. of targeted core per examination^∗^	6 (4-8)	6 (4-8)	0.820
Per examination maximum Gleason score			>0.99
Total	903	261	
Gleason score < 7	464	141	
No prostate cancer	216	58	
3 + 3	248	83	
Gleason score = 7	361	99	
3 + 4	270	71	
4 + 3	91	28	
Gleason score > 7	78	21	
3 + 5	9	0	
4 + 4	35	8	
4 + 5	26	10	
5 + 3	0	1	
5 + 4	7	2	
5 + 5	1	0	
Per systematic core Gleason score			0.119
Total	7083	2025	
Gleason score < 7	6750	1907	
No prostate cancer	6155	1719	
3 + 3	595	188	
Gleason score = 7	275	98	
3 + 4	217	69	
4 + 3	58	29	
Gleason score > 7	58	20	
3 + 5	5	4	
4 + 4	30	2	
4 + 5	16	8	
5 + 3	0	0	
5 + 4	6	6	
5 + 5	1	0	

^∗^Data in parentheses are the interquartile range.

**Table 2:** MIPCa detection performance of WSUNet.

PI-RADS	Training set		Testing set	
	AUC	Sensitivity^∗^	AUC	Sensitivity^∗^
All (1-5)	0.798 (0.775-0.819)	0.817 [272/333] (0.781-0.850)	0.764 (0.728-0.798)	0.797 [94/118] (0.737-0.856)
≥3	0.794 (0.773-0.815)	0.814 [263/323] (0.780-0.848)	0.762 (0.725-0.795)	0.803 [94/117] (0.744-0.863)

Note. Data in parentheses are 95% CIs. AUC = area under the receiver operating characteristic curve. ^∗^Data are percentages, with number of participants in brackets. *PI-RADS* refers to the maximum PI-RADS for all outlined lesions in one examination. Since most of the patient's PI-RAD is greater than 3, so this part of the data has been presented alone. *MIPCa detection* refers to the prediction of systematic biopsy.

**Table 3:** Precision (detection rate) of systematic biopsy and WSUNet.

PI-RADS	Systematic biopsy^∗^	WSUNet^∗^	Ratio	Biopsy saving ratio (number)	*p* value
All (1-5)					
Training set	0.047 [333/7083]	0.090 [272/3040]	1.904	0.475 (3361)	<0.005
Testing set	0.058 [118/2025]	0.111 [94/846]	1.913	0.476 (963)	<0.005
≥3					
Training set	0.047 [323/6902]	0.088 [263/2993]	1.870	0.489 (3226)	<0.005
Testing set	0.059 [117/1954]	0.112 [94/843]	1.893	0.463 (905)	<0.005

Note. ^∗^Data are percentages, with numbers of participants in brackets. *Biopsy saving ratio (number)* refers to the proportion (number) of biopsy cores that can be reduced by the proposed model when the same number of positive cores is detected. Biopsy saving number = all systematic biopsy number–model‐predicted positive number∗(all biopsy‐positive number/model‐predicted true positive number). Biopsy saving ratio = biopsy saving number/all systematic biopsy number. Ratio = the precision of WSUNet/the precision of systematic biopsy. *Precision* refers to the proportion of true positive results among all positive results, which might reflect specific cancer detection rates in retrospective conditions. *PI-RADS* refers to the maximum PI-RADS for all outlined lesions in one examination. Since most of the patient's PI-RAD greater than 3, so this part of the data has been presented alone.

## Data Availability

All data used in this article are derived from public datasets, and proper citations have been included as required.
